# Abdominal and Peripheral Tissue Oxygen Supply during Selective Lower Body Perfusion for the Surgical Repair of Congenital Heart Disease: A Pilot Study

**DOI:** 10.3390/jcdd9120436

**Published:** 2022-12-05

**Authors:** Harry Magunia, Jana Nester, Rodrigo Sandoval Boburg, Christian Schlensak, Peter Rosenberger, Michael Hofbeck, Marius Keller, Felix Neunhoeffer

**Affiliations:** 1 Department of Anesthesiology and Intensive Care Medicine, University Hospital Tuebingen, Eberhard-Karls-University Tuebingen, Hoppe-Seyler-Str. 3, 72076 Tuebingen, Germany; 2 Department of Cardiovascular and Thoracic Surgery, University Hospital Tuebingen, Eberhard-Karls-University Tuebingen, Hoppe-Seyler-Str. 3, 72076 Tuebingen, Germany; 3 Department of Pediatric Cardiology, Pulmonology and Intensive Care Medicine, University Hospital Tuebingen, Eberhard-Karls-University Tuebingen, Hoppe-Seyler-Str. 1, 72076 Tuebingen, Germany

**Keywords:** pediatric cardiac surgery, cardiopulmonary bypass, lower body perfusion, aortic arch surgery, coarctation

## Abstract

Background: Lower body perfusion (LBP) may be a strategy for maintaining organ perfusion during congenital heart disease surgery. It is hypothesized that renal and lower limb oxygen supply during LBP is superior to off-pump surgery and comparable to that of a standard cardiopulmonary bypass (CPB). Methods: in this prospective single-center study, patients aged <1 year were recruited if they were scheduled for a correction of aortic arch anomalies using antegrade cerebral perfusion and LBP (group 1), a repair of coarctation during aortic cross-clamping (group 2), or surgery under whole-body CPB (group 3). Renal (prefix “r”) and peripheral (prefix “p”) oxygen saturation (SO_2_), hemoglobin amount (Hb), blood velocity (Velo), and blood flow (Flow) were measured noninvasively. Results: A total of 23 patients were included (group 1, n = 9; group 2, n = 5; group 3, n = 9). Compared to the baseline values, rSO_2_ and pSO_2_ decreased significantly in group 2 compared to groups 1 and 3. Conversely, rHB significantly increased in group 2 compared to groups 1 and 3, reflecting abdominal venous stasis. Compared to group 3, group 1 showed a significantly lower pFlow during CPB; however, rFlow, pFlow, and pVelo did not differ. Conclusion: according to these observations, LBP results in an improved renal oxygen supply compared to off-pump surgery and may prove to be a promising alternative to conventional CPB.

## 1. Introduction

Maintaining organ perfusion and preserving tissue homeostasis are major goals during a cardiopulmonary bypass (CPB) or the correction of congenital heart disease (CHD). Special entities, such as aortic arch reconstructions (AARs), are associated with other cardiac diseases, such as hypoplastic left heart syndrome, or as a single disease. Due to the site of surgery and the inevitable interruptions of systemic perfusion, special strategies for organ protection are vital to minimize ischemic tissue damage. In recent years, AAR has been performed under deep hypothermic circulatory arrest (DHCA, 20–25 °C), and the preservation of organ integrity has been solely guaranteed by profound hypothermia. Antegrade cerebral perfusion (ACP) has evolved as a novel strategy to avoid circulatory arrest in the most vulnerable organ, the brain. This technique improves cerebral outcomes and allows prolonged surgeries [1]. While ACP primarily maintains the perfusion of the brain, systemic hypothermia is still needed to protect the abdominal organs from ischemic damage, including the kidneys, intestines, and liver. Although several studies have reported decreased lactate levels in patients receiving ACP, the restoration of organ perfusion can lead to reperfusion syndromes (resulting in systemic hypoperfusion, high lactate levels, and acidosis), potentially impairing patient outcomes. In newborns, abdominal hypoperfusion following prolonged lower body circulatory arrest in hypothermia can lead to deleterious effects, including acute kidney injury and necrotizing enterocolitis. Acute kidney injury is a frequent complication following CPB and limits patient prognosis [2,3,4]. Thus, continuous lower body perfusion appears to be a reasonable goal to avoid these complications. Several techniques, including direct descending aortic cannulation, have been published [5]. A technique applying retrograde aortic perfusion using a vascular sheath in the femoral artery has been established at our institution [6]. The monitoring of postoperative renal regional oxygen metabolism and microcirculation using a combination of laser Doppler flowmetry and tissue spectrometry allows for the early prediction of acute kidney injury in infants after on-pump surgery [7]. Schindler et al. found that this method is highly accurate for the detection of even slight changes in flow characteristics and oxygenation supply during surgery using CPB [8].

This method might be able to evaluate and develop different lower body perfusion strategies during CPB. However, data on visceral and lower limb muscular tissue oxygen supply are sparse. This study assessed oxygen supply using an innovative device in neonates and small infants, requiring CPB combined with ACP and LBP for complex aortic arch surgery, off-pump coarctation repair, and standard CPB-based surgery. It is hypothesized that selective LBP results in improved abdominal and lower limb oxygen supply compared with off-pump surgery, with results comparable to those of standard CPB.

## 2. Materials and Methods

### 2.1. Study Design

The study was approved by the Ethics Committee of Eberhard-Karls-University Tuebingen (IRB#573/2012BO1 and amendment of 16 March 2017) and was conducted in accordance with the Declaration of Helsinki. Neonates and infants undergoing aortic arch reconstruction, aortic coarctation (CoA) resection, or other cardiac surgery for CHD using CPB were enrolled in this prospective, single-center, observational pilot study from July 2019 to March 2020. The inclusion criteria were a patient age of <1 year, cardiac surgery performed using CPB or CoA resection without CPB, and written parental informed consent. Demographic data included age at surgery, preoperative weight and height, sex, cardiac diagnosis, surgical procedure, and CPB data. No therapeutic intervention was initiated according to the study protocol.

### 2.2. Patients

Neonates and infants were divided into three groups according to the procedure and the perfusion strategy employed. The main focus was on the study group defined by aortic arch procedures that required ACP combined with LBP. The study group was compared to patients with isolated CoA undergoing off-pump resection with approximately 10–20 min of aortic cross-clamping time and those patients undergoing surgical procedures with conventional CPB and whole-body perfusion, resulting in the following three groups:

Group 1: CPB with ACP and LBP
Group 2: Resection of isolated CoA without CPB
Group 3: Conventional CPB with whole-body perfusion

### 2.3. Anesthesia

All surgeries were performed under general anesthesia. Standardized monitoring and instrumentation were performed, including the placement of an arterial line and a central venous catheter. In cases planned for LBP, a vascular sheath was introduced into the left femoral artery based on the institutional protocol [6]. Age-adjusted goals for mean arterial pressure management were used, and hypotonic phases during CPB were attenuated by continuous noradrenaline infusion. 

### 2.4. Surgical Procedure and Cardiopulmonary Bypass

Surgical repair of isolated CoA was performed without the use of CPB by cross-clamping the aorta between the left carotid and left subclavian arteries after low-dose systemic heparinization (100IU unfractionated heparin per kg body weight). Surgical procedures involving extensive reconstruction of the aortic arch were performed using moderate hypothermia (28–30 °C) and a combination of ACP using a shunt anastomosed to the brachiocephalic trunk, combined with retrograde LBP via the femoral artery sheath. This technique has been described in detail [6]. All other surgical procedures included in this study were performed using standardized CPB via direct aortic and bicaval cannulation combined with moderate-to-mild hypothermia (28–34 °C). During CPB, the target arterial blood pressures were 35–40 mmHg. During CPB, arterial blood gases were measured repeatedly and adjusted based on the pH-stat principle during the hypothermia phases. 

### 2.5. Monitoring of Tissue Oxygen Supply and Microperfusion

For the measurement of oxygen supply and local oxygen consumption in the regions of interest, we used the “oxygen to see” (O2C) device (LEA Medizintechnik, Gießen, Germany), providing good reliability [9]. This is an optical, noninvasive CE-certified method that combines the advantages of white light spectrometry and laser Doppler flowmetry (Figure S1). This allows for the measurement of absolute oxygen saturation (SO_2_), local hemoglobin amount (Hb), blood flow velocity (Velo), and relative blood flow (Flow). The local oxygen metabolism and microperfusion can be evaluated using the O2C method. Tissue spectrometry and laser Doppler flowmetry techniques and their application in neonates and infants have been described in detail in previous studies [7,8,10,11,12,13,14]. The movement of red blood cells causes a Doppler shift in the detected laser light and serves as an indicator of the blood flow velocity. The number of moving red blood cells within the tissue, together with the blood flow velocity, reflects the blood flow. SO_2_ is determined by the color of the blood, which changes according to the degree of oxygen saturation of hemoglobin (unit: percentage). The relative hemoglobin amount (Hb) for the illuminated tissue volume is calculated from the portion of absorbed light. Flow, Velo, and Hb are presented in arbitrary units (AUs), defined by the manufacturer and derived from electrical values of frequencies and amplitudes.

For intraoperative measurement, two different glass fiber probes were used: for the kidney (prefix “r” for renal), the flat probe LF-3-023, with a measuring depth of approximately 1.6–1.8 cm, was placed above the flank, and for peripheral measurement (prefix “p”), a flat probe with a measuring depth of approximately 2–3 mm was attached to the calf (on the opposite leg to the femoral artery sheath).

**Figure 1:**
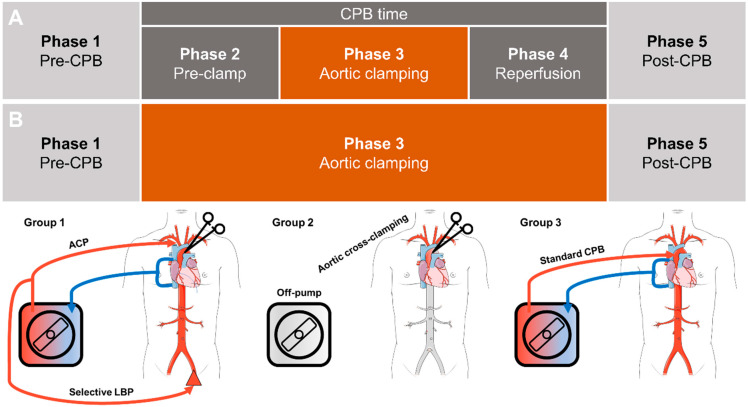
Time points and perfusion strategies of intraoperative tissue oxygen supply measurements. (**A**): standard cardiopulmonary bypass (patient group 3) and selective lower body perfusion (patient group 1) for aortic arch surgery are divided into five phases. (**B**): patients who underwent isolated coarctation resection (group 2) did not undergo CBP, leaving only phases 1, 3 and 5 for intraoperative measurements. ACP: antegrade cerebral perfusion; CPB: cardiopulmonary bypass; LBP: lower body perfusion.

The measurements were performed continuously for each patient during the surgical procedure. Figure 1 provides an overview of the time points. All procedures involving selective perfusion (LBP and ACP) or conventional CPB were divided into five phases (Figure 1A). During CoA correction surgery, these measurements were divided into phases 1, 3, and 5 (Figure 1B).

In addition to continuous O2C measurements, blood gas analyses were performed intermittently every 20–30 min intraoperatively. The lowest intraoperative temperature was also recorded.

### 2.6. Statistical Analyses

PRISM (GraphPad Software, San Diego, CA, USA) was used for the statistical analyses. This prospective observational study used descriptive and inferential statistical procedures with explorative hypothesis generation.

Normal distribution was evaluated using the Shapiro–Wilk test. Continuous data are expressed as medians with interquartile ranges or means with standard deviations. Categorical data were presented as frequencies and percentages. Statistical significance was set at *p* < 0.05. Continuous variables with normal distributions were compared using unpaired *t*-tests, and variables with non-normal distributions were compared using the Mann–Whitney tests. Comparisons of more than two groups were performed using Welch’s analysis of variance (ANOVA), including the post hoc application of Dunnett’s method for multiple comparisons in the case of normal distributions of all included variables; otherwise, Kruskal–Wallis tests, including Dunn’s method, were used. Differences in proportions between the groups were compared with Chi-squared tests. The parameters of noninvasive tissue oxygen supply measurements were normalized to the initial (baseline) values during phase 1 in each patient, reflecting 100 relative percent (rel. %), and comparisons were performed using these relative percentages. 

## 3. Results

### 3.1. Patient Characteristics

A total of 23 neonates and infants were enrolled, and no patients were excluded from the study (the date of surgery for the first patient was 25 July 2019; the date of surgery for the last patient was 4 March 2020). All the patients underwent cardiac surgery for the first time. The patients’ diagnoses in group 1 were hypoplastic left heart syndrome (n = 3), hypoplastic aortic arch with CoA (n = 3), double outlet right ventricle with d-transposition of the great arteries, and aortic arch hypoplasia (n = 1), double inlet left ventricle with d-transposition of the great arteries (n = 1), and common arterial trunk type A4 with interrupted aortic arch (n = 1). Group 2 included five neonates with CoA. Nine patients who underwent surgery with standard CPB were included in group 3. Diagnoses included d-transposition of the great arteries (n = 7), double outlet right ventricle of Fallot type (n = 1) and, CoA with ventricular septal defect and aortic valve hypoplasia (n = 1). The detailed data are provided in Table 1. 

### 3.2. Perfusion Kinetics

**Figure 2:**
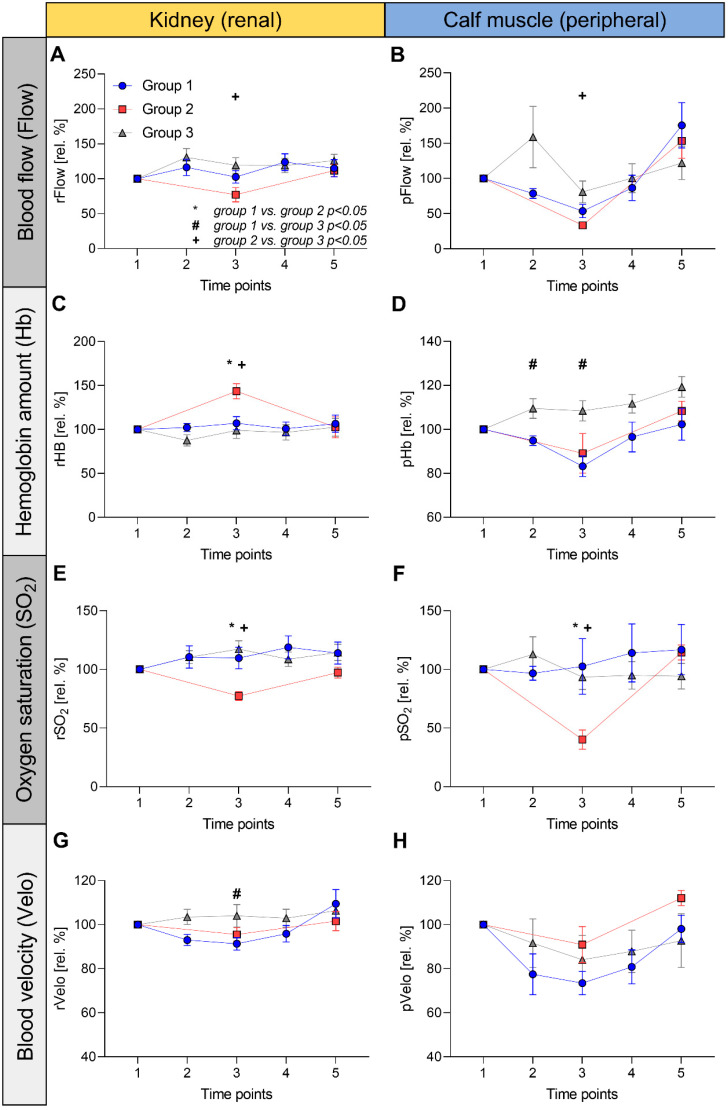
Intraoperative course of tissue oxygen supply parameters of the kidney and calf muscle during selective lower body perfusion. Tissue oxygen supply is assessed noninvasively using the O2C device and yielded blood flow (Flow, (**A**,**B**)), hemoglobin amount (Hb, (**C**,**D**)), oxygen saturation (SO_2_, (**E**,**F**)), blood velocity (Velo, (**G**,**H**)) of the kidney (prefix “r” for renal, left column), and the calf muscle (prefix “p” for peripheral, right column). In each diagram, the values of the patients who underwent cardiac surgery under selective lower body perfusion (group 1), those who underwent off-pump coarctation resection (group 2), and the patients who underwent procedures under standard cardiopulmonary bypass (group 3) are given in relative percentages (rel. %), normalized to the initial values (time point 1) using symbols (mean) and antennas (standard error of the mean). Symbols reflecting statistically significant differences (*p* < 0.05) between the groups: * group 1 vs. group 2, # group 1 vs. group 3, + group 2 vs. group 3.

Figure 2 shows the relative changes in each parameter normalized to the baseline values (at phase 1, reflecting 100%), while Figure S2 shows the curves of the absolute values.

### 3.3. Renal (rFlow) and Peripheral Blood Flow (pFlow)

There was a marked decrease in rFlow at phase 3 in group 2 (Figure 2A) from 100% (230 ± 20 AU), initially to 77 ± 23% (179 ± 60 AU), which is in contrast to group 1 (from 296 ± 150 AU to 318 ± 206 AU or 116 ± 33%, *p* = 0.09) and group 3 (from 203 ± 43 AU to 232 ± 40 AU or 119 ± 34%, *p* = 0.02). All the groups returned to near-baseline levels at time point 5. The pFlow drop (Figure 2B) of group 2 at time point 3 (from 15 ± 4 AU to 5 ± 2 AU or 33 ± 9%) was more pronounced than in group 1 (from 9 ± 8 AU to 4 ± 4 AU or 53 ± 28%, *p* = 0.30) and group 3 (from 11 ± 7 AU to 10 ± 9 AU or 81 ± 46%, *p* = 0.38). Group 1 (to 13 ± 13 AU or 175 ± 35%) and group 2 (to 24 ± 11 AU or 153 ± 122%) showed dramatically increased pFlow at the end of surgery compared to the preoperative phase.

### 3.4. Renal (rHb) and Peripheral Hemoglobin Amount (pHb)

The rHb of Group 2 (Figure 2C) increased from 83 ± 22 AU at baseline to 118 ± 23 AU (143 ± 19%) during aortic cross-clamping (phase 3), whereas the rHb of the other two groups remained constant during CPB (group 1: from 76 ± 14 AU to 81 ± 18 AU or 108 ± 22%, *p* = 0.01; group 3: from 81 ± 28 AU to 75 ± 13 AU or 99 ± 27%, *p* > 0.01). In contrast, calf pHb in group 2 decreased from 51 ± 14 AU to 43 ± 8 AU (89 ± 20%) during phase 3 (Figure 2D). LBP (group 3) resulted in a similar course of pHb to group 1 (decreased to 83 ± 14% at time point 3). Overall, the changes in pHb were moderate within the groups.

### 3.5. Renal (rSO_2_) and Peripheral Oxygen Saturation (pSO_2_)

The rSO_2_ and pSO_2_ showed a comparably constant course throughout the measurements in groups 1 and 3 (Figure 2E,F). In contrast, group 2 showed a considerable decrease in both rSO_2_ (from 62 ± 9% to 48 ± 8% or 77 ± 8 rel. %) and pSO_2_ (from 80 ± 18% to 30 ± 12% or 40 ± 18 rel. %) at time point 3.

### 3.6. Renal (rVelo) and Peripheral Blood Velocity (pVelo)

The rVelo showed no notable changes within the groups or among each other. During phase 3, pVelo was most pronounced in the LBP group (from 12 ± 2 AU to 9 ± 2 AU or 73 ± 16%) compared to the other groups (group 2: from 12 ± 1 AU to 11 ± 2 AU or 91 ± 18%, *p* = 0.11; group 3: from 11 ± 3 AU to 10 ± 4 AU or 84 ± 33%, *p* = 0.40). Only group 2 returned to above baseline pVelo values during phase 5 (to 13 ± 1 AU or 112 ± 8%).

### 3.7. Between-Group Comparisons of rHb and rSO_2_

Measurements of rSO_2_ in the clamp phase (time point 3) showed significant differences between the groups (Figure 2E). Patients undergoing CoA resection (group 2) showed significantly lower rSO_2_ than group 3 (77 ± 8 rel. % vs. 117 ± 21 rel. %, *p* < 0.001). There was also a significant difference between groups 2 and 1 (110 ± 27 rel. %, *p* < 0.01). No significant difference was observed between the LBP and whole-body CPB groups (*p* = 0.52). Hence, selective LBP resulted in maintained rSO_2_ compared with conventional CPB, in contrast to off-pump CoA resection.

There were further clear differences in rHb between the groups during the clamping of the aorta (Figure 2C). Group 2 showed significantly higher values than group 3 (144 ± 19 rel. % vs. 99 ± 27 rel. %, *p* < 0.01), and the same relationship was observed between groups 2 and 1 (108 ± 22 rel. %, *p* = 0.01). No significant differences were found between the CPB groups. As a surrogate for venous stasis, the course of rHb appears to reflect sufficient microperfusion in the LBP-based CPB strategy.

In summary, these findings indicate that renal tissue oxygen supply is significantly impaired during a circulatory arrest for CoA resection; however, it can be preserved using selective LBP.

### 3.8. Laboratory Values

Compared with baseline values, a marked elevation in intraoperative lactate levels was observed in all patient groups (Table 2). In the CPB groups (groups 1 and 3), lactate levels were highest upon intensive care unit (ICU) admission and normalized within 24 h. There was no difference between the lactate levels of groups 1 and 3 at ICU admission; however, both groups had higher lactate levels than group 2. Neither lactate dehydrogenase nor creatinine levels differed significantly between the groups throughout the perioperative period. Both CPB groups (groups 1 and 3) showed significantly elevated aspartate aminotransferase (ASAT) levels at ICU admission compared with patients undergoing off-pump CoA resection. The difference in ASAT between groups 1 and 2 remained significant after 24 h.

### 3.9. Clinical Course

During ICU therapy, prolonged and complicated treatment was observed in two patients in group 1, both of whom had hypoplastic left heart syndrome. These patients required venoarterial extracorporeal membrane oxygenation due to postoperative low cardiac output syndrome. One patient required renal replacement therapy and died during ICU treatment.

## 4. Discussion

State-of-the-art cardiac surgery is only possible due to the paramount advances in perfusion strategies and novel systems related to cardiopulmonary bypass. In the early years of surgical correction of congenital heart disease, hypothermia, and circulatory arrest were the only procedures used for organ protection. Currently, reconstructive surgery of the aortic arch and Norwood-type surgeries are performed using CPB and ACP for brain protection in many centers. Structures below the aortic arch hereby still undergo a phase of severe hypoperfusion, and organ protection should be ensured by hypothermia [15]. However, protection via hypothermia can be inadequate, and together with a rewarming injury, it may result in severe organ damage. Therefore, innovative strategies to maintain blood flow and tissue oxygenation in all body regions may be beneficial.

This study compared noninvasively-assessed tissue oxygen supply to the kidney and calf muscles in three common settings of surgical correction of CHD. As expected, tissue perfusion was maintained during standard CPB, whereas renal and peripheral blood flow and oxygen saturation were significantly reduced during aortic cross-clamping for coarctation repair. These data further indicate that the tissue oxygen supply reflected by SO_2_ is maintained by selective lower body perfusion with vascular sheaths during aortic arch reconstruction. This translates into similar courses of laboratory results between selective LBP and standard CPB in terms of systemic perfusion (lactate, LDH) and hepatic function (ASAT). Overall, this study’s hypothesis was confirmed by the results.

The development of congenital cardiac surgery is accompanied by improvements in body perfusion strategies and the protection of organ integrity. Postoperative organ dysfunction is associated with poor outcomes. Acute kidney injury after congenital cardiac surgery leads to an extended ICU stay and the prolonged duration of mechanical ventilation [16]. Despite a less profound body of data, postoperative liver dysfunction is also associated with impaired prognosis [17,18]. Currently, the protection of abdominal organs in infants undergoing aortic arch surgery is either performed by DHCA or by a strategy combining antegrade cerebral perfusion for brain protection combined with hypothermia [19]. Various studies have compared the results of these two techniques in terms of neurological function and acute kidney injury, in part with conflicting results [20,21,22]. Therefore, the preservation of blood flow and oxygen delivery to the lower body, including the vital abdominal organs, is of high clinical relevance. Several technical approaches have been proposed for LPB during on-pump aortic arch surgery [6,23,24]. Clinical observational trials or case series have demonstrated that LBP is associated with reduced intraoperative lactate levels and a lower incidence of acute kidney and/or hepatic injury [6,24,25,26,27,28]. Similar observations were made using vascular sheaths and catheters introduced into the femoral artery for LBP [6,28]. As the diameter of the catheters is limited by the femoral artery, only a low blood flow of approximately 30 to 50 mL/min can be administered. Promising clinical results have shown that sufficient organ protection is possible with this technique. The noninvasive monitoring of renal microperfusion used in the present study may further provide improved prediction of postoperative kidney injury following non-cpb surgery [29], which needs to be investigated in future trials. To the best of our knowledge, this is the first study to report data on in vivo microperfusion and tissue oxygen supply for the evaluation of the effects of LBP on organ perfusion.

Conventional CoA repair performed without cardiopulmonary bypass served as a model for changes in tissue perfusion due to circulatory arrest in the lower body. Our microperfusion studies showed reversible immediate blood flow cessation and oxygen desaturation during aortic cross-clamping and unclamping. CBP with whole-body perfusion served as the second model to assess tissue perfusion in the lower body. In our study, only small variations in blood flow, Hb, and oxygen saturation were observed during CPB; therefore, adequate oxygen delivery to all tissues was likely. Likewise, CPB with ACP and LBP resulted in a maintained tissue oxygen supply, as assessed by noninvasive tissue oxygenation and laboratory results.

The present study has several limitations. First, it was a single-center study that included only a small number of patients. Larger multicenter trials are warranted to confirm these findings. Second, this study lacked a comparison with infants undergoing aortic arch surgery with ACP only. This should be assessed in a prospective randomized trial to investigate the isolated effect of selective LBP. Third, the present data show changes in tissue oxygen supply and the laboratory values as surrogates for short-term organ dysfunction. As mid- and long-term organ functions are of great importance, future studies should assess long-term changes to determine whether LBP has an additional prognostic value. Furthermore, the O2C method quantifies oxygen supply and microperfusion only in distinct regions, and the results may reflect the perfusion of the entire organ. The current approach does not allow for the separate regulation of ACP and LBP blood flows because both are controlled by the same pump.

In summary, a simple sheath-based approach for LBP leads to a sufficient tissue oxygen supply to the lower body and has the potential to provide adequate organ protection during complex aortic arch surgery and Norwood-type procedures.

## Figures and Tables

**Table 1:** Patient characteristics and intraoperative data.

	Group 1 CPB with ACP and LBP	Group 2 CoA Resection	Group 3 Standard CPB	*p* Value
N	9	5	9	-
Age (days)	14 (IQR 10 to 48)	7 (IQR 6 to 89)	11 (IQR 7.5 to 14.5)	0.54
Sex (n, %)				
Male Female	5 (56%) 4 (44%)	3 (60%) 2 (40%)	7 (78%) 2 (22%)	ns
Weight (kg)	3.6 (IQR 3.1 to 3.75)	3.3 (IQR 2.75 to 5.9)	3.8 (IQR 3.4 to 4)	0.32
BSA (m^2^)	0.22 (IQR 0.195 to 0.23)	0.22 (IQR 0.22 to 0.26)	0.22 (0.22 to 0.24)	0.56
Diagnoses (n, %)				
HLHS Aortic arch hypoplasia +/− other defects DILV + TGA CoA TGA (+/− VSD) DORV CoA + VSD	3 (33%) 5 (56%) 1 (11%) - - - -	- - - 5 (100%) - - -	- - - - 7 (78%) 1 (11%) 1 (11%)	- - - - - - -
Intraoperative and CPB data				
CPB time (min)	150 (IQR 101 to 178)	-	154 (IQR 151 to 183)	0.13
Aortic cross clamp time (min)	82 (IQR 64 to 134)	-	130 (IQR 107 to 175)	0.06
Lower body perfusion time (min)	47 (IQR 41 to 74)	-	-	-
Reperfusion time (min)	34 (IQR 14 to 63)		15 (IQR 12 to 20)	0.14
Lowest intraoperative temperature (°C)	31.3 (IQR 30 to 31.9)	37.3 (IQR 36.1 to 38)	33.7 (IQR 31.5 to 35)	<0.001 ^a^

Significant differences for multiple comparisons between groups are displayed as follows: ^a^ group 1 vs. group 2; ns: no significant differences in the proportions between the groups. ACP: antegrade cerebral perfusion; BSA: body-surface area; CoA: aortic coarctation; CPB: cardiopulmonary bypass; DILV: double inlet left ventricle; DORV: double outlet right ventricle; HLHS: hypoplastic left heart syndrome; IQR: inter-quartile range; LBP: lower body perfusion; TGA: transposition of the great arteries; VSD: ventricular septal defect.

**Table 2:** Laboratory results.

	Lactate (mmol/L)				Creatinine (mg/dL)			LDH			ASAT		
	Pre-CPB	CPB	ICU adm.	ICU 24 h	Pre-CPB	ICU adm.	ICU 24 h	Pre-CPB	ICU adm.	ICU 24 h	Pre-CPB	ICU adm.	ICU 24 h
Group 1 (CPB with ACP and LBP)	0.9 ± 0.5	2.0 ± 1.2	3.4 ± 1.7	1.6 (1.4 to 2)	0.5 ± 0.2	0.5 ± 0.2	0.5 (0.5 to 0.8)	326 ± 54	455 ± 208	406 (296 to 614)	31 (23 to 48)	136 ± 71	58 (39 to 157)
Group 2 (off-pump CoA Resection)	0.9 ± 0.4	2.0 ± 0.7	1.5 ± 0.5	1.1 ± 0.4	0.4 ± 0.1	0.4 ± 0.1	0.4 ± 0.1	383 ± 113	327 ± 84	335 ± 75	49 ± 26	46 ± 22	30 ± 4
Group 3 (Standard CPB)	0.9 ± 0.4	1.7 ± 0.8	2.9 ± 0.9	1.4 ± 0.5	0.5 ± 0.2	0.4 ± 0.2	0.5 ± 0.2	336 ± 68	491 ± 192	371 ± 160	31 ± 17	130 ± 38	43 (38 to 88)
*p* value	0.94	0.56	**<0.01** ^ab^	0.13	0.28	0.46	0.13	0.66	0.05	0.49	0.26	**<0.001** ^ab^	**0.02** ^a^

Significant differences for multiple comparisons between the groups are displayed as follows: ^a^ group 1 vs. group 2, ^b^ group 2 vs. group 3. Adm.: admission; ASAT: aspartate aminotransferase; CoA: aortic coarctation CPB: cardio-pulmonary bypass; ACP: antegrade cerebral perfusion; LBP: lower body perfusion; LDH: lactate dehydrogenase.

## Data Availability

All data can be shared by the authors upon reasonable request and in accordance with German privacy regulations.

## Supplementary Materials

The following supporting information can be downloaded at: https://www.mdpi.com/article/10.3390/jcdd9120436/s1, Figure S1: The technique uses a combination of laser Doppler flowmetry and white light spectroscopy to quantify absolute oxygen saturation (SO_2_), local hemoglobin amount (_rel_Hb), the blood flow velocity (Velocity), and the relative blood flow (Flow). Figure S2: Intraoperative course of tissue oxygen supply parameters of the kidney and calf muscle during selective lower body perfusion (absolute values). Tissue oxygen supply is assessed non-invasively using the O2C device and yielded blood flow (Flow, A,B), hemoglobin amount (Hb, C,D), oxygen saturation (SO_2_, E+F), and blood velocity (Velo, G,H) of the kidney (prefix “r” for renal, left column) and the calf muscle (prefix “p” for peripheral, right column). In each diagram, the values of patients undergoing cardiac surgery under selective lower body perfusion (group 1), those undergoing off-pump coarctation resection (group 2) and patients undergoing procedures under standard cardiopulmonary bypass (group 3) are given in the corresponding measurement units using symbols (mean) and antennas (standard error of the mean).

## Abbreviations

AAR	aortic arch reconstruction
ACP	antegrade cerebral perfusion
ASAT	aspartate aminotransferase
AU	arbitrary unit(s)
CPB	cardiopulmonary bypass
CHD	congenital heart disease
DHCA	deep hypothermic circulatory arrest
Flow	Relative blood flow quantified by the O2C device
Hb	Relative hemoglobin amount quantified by the O2C device
ICU	intensive care unit
LBP	lower body perfusion
p	peripheral
r	renal
SO_2_	oxygen saturation of hemoglobin quantified by the O2C device
Velo	blood flow velocity quantified by the O2C device
