# Supplementary material for: Abdominal and Peripheral Tissue Oxygen Supply during Selective Lower Body Perfusion for the Surgical Repair of Congenital Heart Disease: A Pilot Study
Source: J Cardiovasc Dev Dis. 2022 Dec 5;9(12):436. doi: 10.3390/jcdd9120436 (PMC9782002; doi:10.3390/jcdd9120436)
Supplement: Supplementary file 1 [file jcdd-09-00436-s001.zip › jcdd-2056823-supplementary.pdf]

**Figure S1:** **Schematic display of the "oxygen to see" method for assessment of tissue oxygen supply**

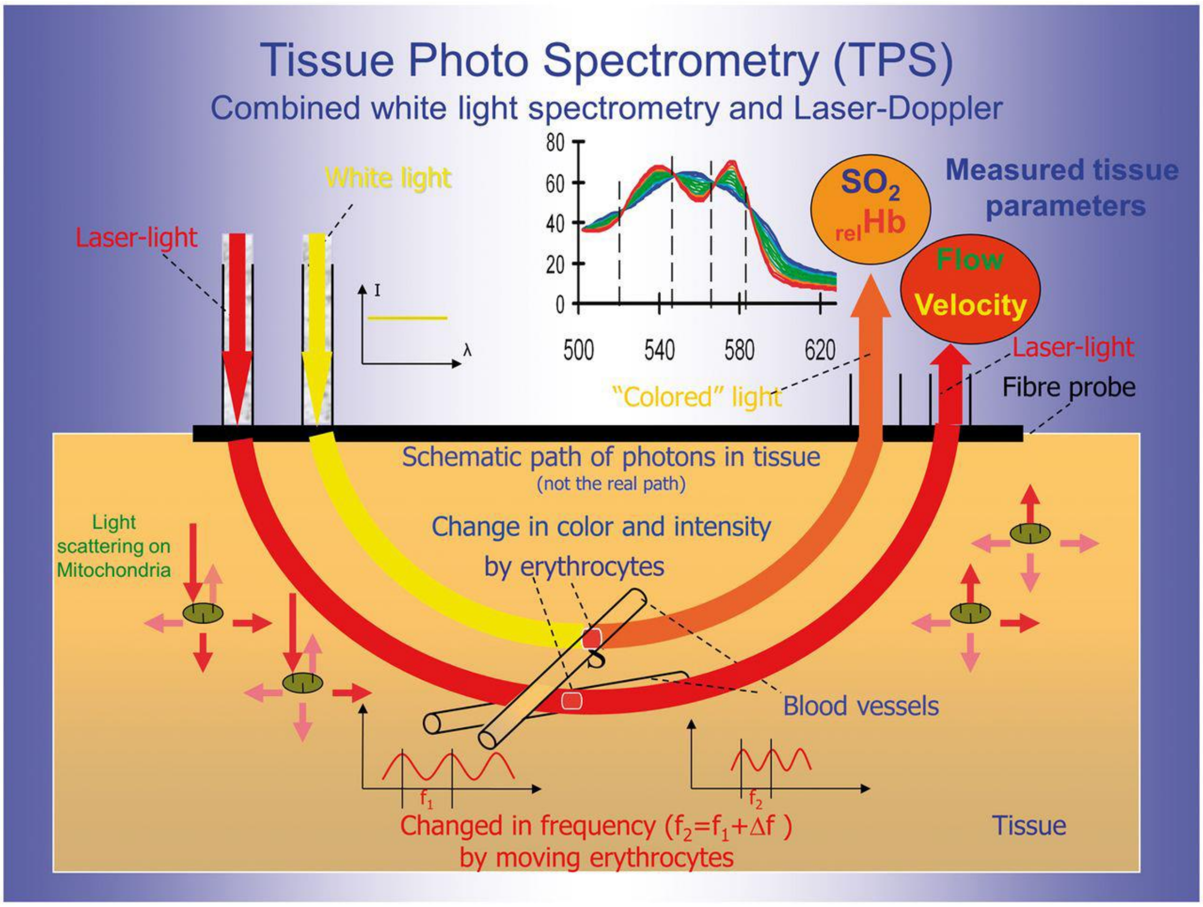

**Figure S2:** **Intraoperative course of tissue oxygen supply parameters of the kidney and calf muscle (absolute values)**

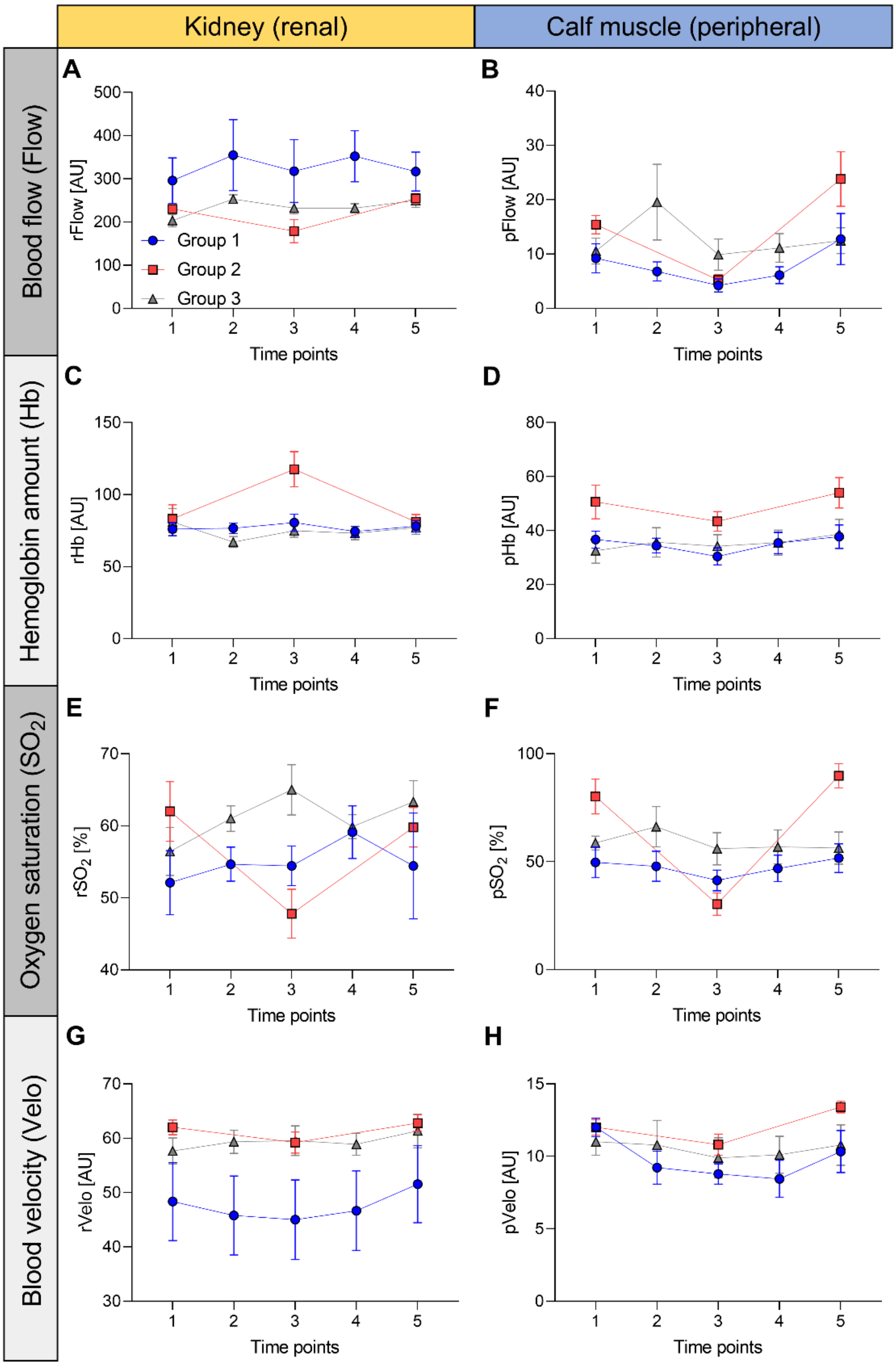


Tissue oxygen supply is assessed non-invasively using the O2C device and yielded blood flow (Flow, A+B), hemoglobin amount (Hb, C+D), oxygen saturation ($SO_2$, E+F), and blood

velocity (Velo, G+H) of the kidney (prefix “r” for renal, left column) and the calf muscle (prefix “p” for peripheral, right column). In each diagram, the values of patients undergoing cardiac surgery under selective lower body perfusion (group 1), those undergoing off-pump coarctation resection (group 2) and patients undergoing procedures under standard cardiopulmonary bypass (group 3) are given in the corresponding measurement units using symbols (mean) and antennas (standard error of the mean).
